# 1176. Experience with PCV10 Implementation in Colombia and More Severe Course of Pneumococcal Pneumonia in children: A Multicenter Study, 2008 – 2019 (Neumocolombia Network)

**DOI:** 10.1093/ofid/ofab466.1369

**Published:** 2021-12-04

**Authors:** Ivan Felipe Gutiérrez Tobar, Juan Pablo Londoño, Cristina Mariño Drews, Sandra Beltran, Aura Lucia Leal Castro, Aura Lucia Leal Castro, Jaime alberto Patiño-Niño, Martha Isabel Alvarez-Olmos, Rocio Barrero Barreto, Fabio Espinosa, Maria Alejandra Suarez, Nicolas Ramos, Vivian Marcela Moreno Mejia, Alejandra Marin, Claudia Rocio Sierra Parada, Angela Pescador

**Affiliations:** 1 Clinica Infantil Colsubsidio, Clínica Infantil Santa María del Lago, Bogotá, Distrito Capital de Bogota, Colombia; 2 Clínica Infantil Colsubsidio, Bogotá, Distrito Capital de Bogota, Colombia; 3 Hospital Militar Central, Bogotá, Distrito Capital de Bogota, Colombia; 4 Clínicas Colsanitas, Bogotá, Distrito Capital de Bogota, Colombia; 5 Grupo para el control de la resistencia bacteriana en Bogotá, GREBO, Bogotá, Colombia, Universidad Nacional de Colombia, Bogotá, Colombia, . Asociación Colombiana de Infectología – Capítulo central, Bogotá, Colombia, Bogotá, Distrito Capital de Bogota, Colombia; 6 Universidad Nacional de Colombia, Bogotá, Distrito Capital de Bogota, Colombia; 7 Fundación Valle de Lili - Universidad ICESI, Cali, Valle del Cauca, Colombia; 8 Fundación Cardio Infantil - Instituto de Cardiología, Bogotá, Distrito Capital de Bogota, Colombia; 9 Hospital Santa Clara - Hospital Universitario Clínica San Rafael, Bogotá, Distrito Capital de Bogota, Colombia; 10 Fundación Hospital Universitario Infantil de San José, Bogotá, Distrito Capital de Bogota, Colombia; 11 Hospital el Tunal, Bogotá, Distrito Capital de Bogota, Colombia; 12 Los Cobos Medical Center, Bogotá, Distrito Capital de Bogota, Colombia; 13 Red Neumocolombia, Bogotá, Distrito Capital de Bogota, Colombia

## Abstract

**Background:**

Pneumococcal conjugate vaccines (PCV) have decreased pneumonia in children. Colombia introduced massive vaccination with PCV10 in 2012.

**Methods:**

Pneumococcal pneumonia cases from 10 hospitals part of an active surveillance network for invasive pneumococcal disease were included. Two periods were compared, pre-PCV10: 2008-2012 and post-PCV10: 2014-2019. The objective was to compare characteristics and outcomes before and after PCV10.

**Results:**

370 cases were included. Serotype 1(15, 11.2%) and 14 (33, 24.6%) were the most frequent in Pre-PCV10, with only 4(3%) 19A and 1(0.7%) serotype 3. Post-PCV10, serotype 1 decreased to 6(3.1%), 14 to 15(7.8%), while 19A increased to 58(30.2%), serotype 3 to 32(16.7%) and 6A to 7(3.6%) (p = < 0.001), (Graph 1). Complicated pneumonia (CN) also increased (13.4% to 31,8%) (p< 0,001). Pre-PVC10, 44% of CN were due to PCV10 serotypes; with no PCV13 serotypes cases. Post-vaccine period, PCV10 explained only 8.2% and PCV13 60.6%(p < 0.001) of CN. Comparing PICU requirement among predominant serotypes on each period; 23.5% of serotypes 14 and 27.2% of serotypes 1 were admitted, while 59.4% of serotypes 3, 56.9 % of 19A and 42.8% of 6A required PICU. The median of hospitalization increased from 8(5.5-15) to 12 (7-22) days (p < 0.001), as well as the frequency of PICU, 32.8% to 51.6 %, (p = 0.001). Penicillin prescription was similar (17.2% -15.7%), with decrease in ampicillin use (28.4% - 3.6%) and increase ampicillin-sulbactam (0.7% to 24%), and ceftriaxone / clindamycin (0.7% to 5.7%) in post-PCV10. The duration of empirical antibiotic treatment was 7(4-11) and increased to 10(6-17) (p = < 0.001). Lethality showed a slight, non-significant increase between periods 7.5% vs. 9.9% (p = 0.57). (Table1)

Graph 1. Serotype distribution 2008 - 2019

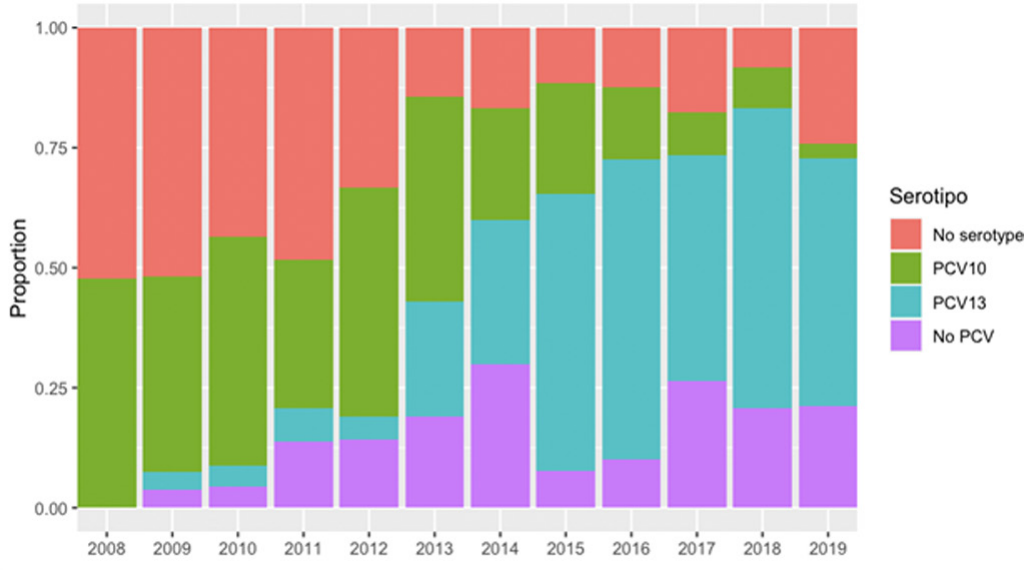

Year 2012, PCV10 introduced 2 + 1 schedule.

Table 1. Outcomes in the Pre-PCV10 and Post-PCV10 Period

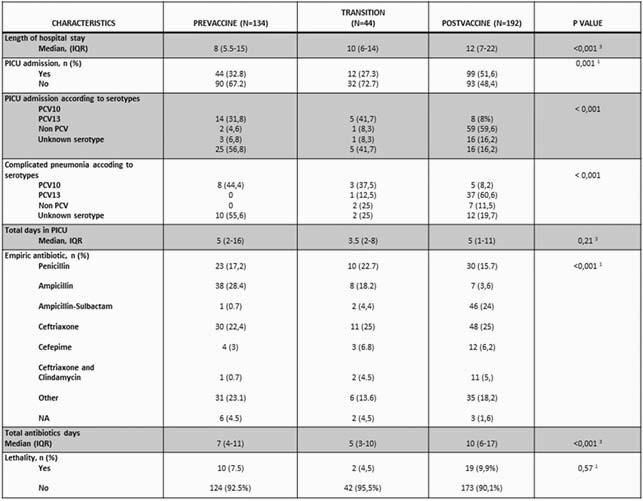

**Conclusion:**

PCV10 significantly decreased vaccine serotypes, with increase in PCV13 serotypes. 19A, 3 and 6A the predominant serotypes had greater severity including PICU admission, CN and more resistance, with an increase in the use of broad-spectrum antibiotics and longer hospitalization. The current data support national and regional evidence on the importance of replacing PCV10 to a higher valence that include 19A, as PCV13, with the aim of reducing the circulation, particularly of this serotype.

**Disclosures:**

**Ivan Felipe Gutiérrez Tobar, n/a**, **Pfizer and MSD** (Advisor or Review Panel member, Research Grant or Support, Speaker’s Bureau, Has received support from Pfizer and MSD for participation in congresses and has received conference payments from Pfizer)**Pfizer and MSD** (Speaker’s Bureau, Other Financial or Material Support, Has received support from Pfizer for participation in congresses) **Cristina Mariño Drews, n/a**, **Pfizer** (Other Financial or Material Support, Has received support from Pfizer for participation in congresses) **Sandra Beltran, n/a**, **Pfizer** (Other Financial or Material Support, Has received support from Pfizer for participation in congresses) **Aura Lucia Leal Castro, MD**, **Pfizer and MSD** (Research Grant or Support, Speaker’s Bureau, Other Financial or Material Support, Has received support from Pfizer for participation in congresses) **Aura Lucia Leal Castro, n/a**, **Pfizer and MSD** (Research Grant or Support, Speaker’s Bureau, Other Financial or Material Support, Has received support from Pfizer for participation in congresses) **Jaime alberto Patiño-Niño, n/a**, **Pfizer** (Research Grant or Support, Speaker’s Bureau, Other Financial or Material Support, Has received support from Pfizer for participation in congresses) **Martha Isabel Alvarez-Olmos, n/a**, **Pfizer** (Other Financial or Material Support, Has received support from Pfizer for participation in congresses) **Rocio Barrero Barreto, n/a**, **Pfizer and MSD** (Other Financial or Material Support, Has received support from Pfizer and MSD for participation in congresses and has received conference payments from Pfizer) **Fabio Espinosa, n/a**, **MSD** (Research Grant or Support, Other Financial or Material Support, Has received support from MSD for other research.) **Nicolas Ramos, n/a**, **Pfizer** (Other Financial or Material Support, Has received support from Pfizer for participation in congresses) **Vivian Marcela Moreno Mejia, n/a**, **Pfizer** (Research Grant or Support)

